# Sex-specific association between gut *Faecalibacterium prausnitzii* and hypertension in male individuals

**DOI:** 10.3389/fmicb.2025.1683587

**Published:** 2025-12-17

**Authors:** Jing Fu, Guosen Bu, Salamaiti Aimaier, Yuchun Yang, Zhen Bao, Muhuyati Wulasihan

**Affiliations:** 1 Department of General Cardiology, The First Affiliated Hospital of Xinjiang Medical University, Urumqi, China; 2 Department of Neurology, The First Affiliated Hospital of Xinjiang Medical University, Urumqi, China; 3 Department of Heart Failure, The First Affiliated Hospital of Xinjiang Medical University, Urumqi, China

**Keywords:** hypertension, gut microbiome, *Faecalibacterium prausnitzii*, mediation analysis, sex differences

## Abstract

**Objective:**

While gut microbiota (GM) dysbiosis has been implicated in hypertension, the sexassociated microbial signatures and their underlying mechanisms remain poorly understood, particularly in populations living in unique geographical and climatic conditions.

**Design:**

Through an integrated approach combining 16S rRNA sequencing, shotgun metagenomics, and serum metabolomics, we systematically investigated the sex-associated characteristics of the gut microbiota in hypertension among a cohort of 200 participants from Xinjiang.

**Methods:**

An initial cohort analysis identified *Faecalibacterium* as a male-associated biomarker for hypertension. Subsequent species-level characterization revealed that *Faecalibacterium prausnitzii (F. prausnitzii)* showed significant negative correlations with systolic blood pressure (SBP). This male-specific association was consistently observed across both 16S rRNA sequencing and shotgun metagenomic datasets. Then, our integrated analysis suggested a potential pathway through which *F. prausnitzii* may be linked to systolic blood pressure in male individuals, with N-phenylacetylglutamine (PAGln) identified as a potential mediating metabolite.

**Conclusion:**

Our study establishes a microbe–metabolite–clinical trait axis in the pathophysiology of sex-associated hypertension and significantly advances our understanding of sex-driven host–microbe interactions.

## Introduction

Hypertension remains one of the most significant modifiable risk factors for cardiovascular and cerebrovascular diseases, contributing to global morbidity and mortality. The global prevalence of hypertension among adults aged 30–79 years was 32% (95% CrI 30–34) in women and 34% (32–37) in men in 2019 (NCD Risk Factor Collaboration, 2021). Genetic predisposition, lifestyle factors (e.g., diet and physical inactivity), and environmental exposures (e.g., air pollution) are well-established contributors to hypertension pathogenesis (Fuchs and Whelton, 2020; Karppanen and Mervaala, 2006). Recently, emerging evidence has suggested that gut microbiome dysbiosis may play a critical role in blood pressure (BP) regulation (Gomez-Arango et al., 2016; Richards et al., 2017; Wilck et al., 2017; Yan et al., 2017).

Mounting evidence highlights that host-associated factors, including ethnicity, geography, diet, and sex, collectively shape gut microbiota (GM) composition and function (Cheng et al., 2021; Su et al., 2023; Zhang et al., 2021), with profound implications for metabolic disease susceptibility (Gao et al., 2021; Kaliannan et al., 2018). Ethnic differences in the GM are linked to genetic ancestry and cultural practices, while geographic factors (e.g., altitude, climate) drive microbial adaptations to local environments (Li et al., 2018; Litchman, 2025). These stratified effects underscore the necessity of subgroup-specific analyses. Ethnicity–geography–diet interactions may obscure sex-associated GM signatures if populations are not appropriately stratified in studies. A previous study reported distinct gut microbial signatures in female hypertensive patients within Hong Kong cohorts (Virwani et al., 2023); however, the generalizability of these findings to other geographic populations remains to be elucidated.

The integration of multi-omics approaches is essential for advancing our understanding of the complex interplay between the gut microbiota and host physiology. While 16S rRNA sequencing provides valuable insights into microbial community structure at the genus level for rapid screening, shotgun metagenomics overcomes this limitation by enabling species-level identification and functional gene annotation (Knight et al., 2018). However, shotgun metagenomics alone cannot capture the downstream metabolic consequences of microbial activity. This gap is addressed by serum metabolomics, which quantifies host–microbe co-metabolites that directly mediate physiological effects (Shaffer et al., 2017).

To address these gaps, we conducted a sex-balanced study in a Xinjiang cohort of 200 participants. We compared the microbial composition of fecal samples using 16S rRNA sequencing and shotgun metagenomics at both genus and species levels. We hypothesized that the relationship between blood pressure and the gut microbiota is sex-specific. Furthermore, we aimed to construct a sex-associated microbe–metabolite–clinical trait axis using a multi-omics approach.

## Methods

### Ethics statement

This study was approved by the Ethics Committee of the First Affiliated Hospital of Xinjiang Medical University (Approval No. K202412-25), and written informed consent was obtained from all participants.

### Participants and sample collection

This study enrolled 100 patients with primary hypertension who were hospitalized at the First Affiliated Hospital of Xinjiang Medical University between February 2024 and June 2024, along with 100 healthy controls, matched for age, sex, and body mass index (BMI), who were recruited from the Health Examination Center of Xinjiang Medical University during the same period. Participants in the hypertension group were required to meet all of the following criteria: (1) a diagnosis of hypertension, defined as systolic blood pressure (SBP) of ≥130 mm Hg or diastolic blood pressure (DBP) of ≥80 mm Hg, based on the 2017 ACC/AHA guidelines (Colantonio et al., 2018); (2) age between 18 and 80 years; (3) a documented family history of continuous residence in Xinjiang for at least three generations; and (4) self-reported regular bowel and bladder habits. Similarly, normotensive control participants were enrolled if they met all of the following criteria: (1) SBP of <120 mm Hg and DBP of <80 mm Hg; (2) age between 18 and 80 years; (3) multigenerational residency in Xinjiang; and (4) regular bowel and bladder habits. Participants were excluded from the study if they met any of the following criteria: (1) current pregnancy or lactation; (2) a prior diagnosis of secondary hypertension; (3) a history of major comorbidities, including malignancy, acute inflammation, coronary heart disease, heart failure, diabetes, renal failure, stroke, peripheral artery disease, or chronic inflammatory diseases; (4) use of medications known to influence the gut microbiota or blood pressure, such as antibiotics, probiotics, prebiotics, laxatives, traditional Chinese herbs, acid-suppressants, immunosuppressants, or antihypertensive drugs, within the 3 months preceding enrollment; or (5) a history of gastrointestinal surgery.

Blood pressure was measured by a trained physician or nurse using a calibrated mercury sphygmomanometer and an appropriately sized cuff. Measurements were taken in the morning after the participants had rested in a seated position for at least 15 min. Following this rest period, three consecutive readings were taken on the same arm at 5-min intervals. The average of these three readings was calculated and used as the final blood pressure value for all subsequent analyses.

All participants provided approximately 2 g of fresh fecal samples, which were immediately transferred and stored at −80 °C for subsequent analysis. Following a 12-h fasting period, 5 mL of peripheral venous blood was collected from each patient. The blood samples were allowed to stand at room temperature for at least 30 min before being centrifuged at 3,000 rpm for 15 min to obtain serum samples, which was then stored at −80 °C. The serum samples were subjected to untargeted metabolomic sequencing for subsequent analysis.

### DNA preparation and sequencing

Genomic DNA was extracted from all samples according to a modified protocol provided in the QIAamp DNA mini kit. 16S rRNA V4 genes of distinct regions were amplified using specific primer (515F-806R, Illumina MiSeq) with the barcode and were measured using Illumina MiSeq. Whole-metagenome shotgun paired-end DNA libraries (insert size 350 bp, read length 150 bp) were constructed according to the manufacturer’s instructions (Illumina, United States) and sequenced using the Illumina HiSeq3000 platform.

### 16S rRNA sequencing analysis

Sequence denoising was conducted using DADA2 (Callahan et al., 2016) in QIIME2 (Bolyen et al., 2019) to generate amplicon sequence variants (ASVs). Species annotation was performed using the SILVA database in QIIME2. To analyze phylogenetic relationships and differences in dominant species across the samples, multiple sequence alignment was performed. The absolute abundance of ASVs was normalized to the sample with the fewest sequences. Subsequent alpha and beta diversity analyses were conducted on the normalized data.

### Shotgun sequencing analysis

fastp (Chen, 2023) was used for preprocessing raw data from the Illumina sequencing platform to obtain clean data for subsequent analysis. Paired reads were discarded under the following conditions: If either read contained adapter contamination, if either read contained more than 10% uncertain nucleotides, or if either read contained more than 50% low-quality nucleotides (base quality less than 5). To account for potential host contamination in the samples, clean data were aligned to the host genome database to filter out reads of host origin. Bowtie 2 (Langmead and Salzberg, 2012) was used with default settings and the following parameters: --end-to-end, −-sensitive, -I 200, and -X 400.

We performed microbial taxonomic profiling and functional annotation using MetaPhlAn4 (Blanco-Miguez et al., 2023). For taxonomic classification, quality-filtered metagenomic reads were aligned to the MetaPhlAn marker database using Bowtie 2 with default parameters. Microbial abundances were estimated based on the number of reads mapped to clade-specific markers and normalized to copies per million (CPM). Functional potential was inferred using HUMAnN 3.0 by mapping reads to the UniRef90 protein database via DIAMOND (Buchfink et al., 2021).

### Untargeted metabolomics

Serum metabolomic analysis was conducted by Novogene Co., Ltd. (Beijing, China) using ultra-high-performance liquid chromatography–tandem mass spectrometry (UHPLC–MS/MS). Samples were prepared by protein precipitation with ice-cold 80% methanol, followed by vortex mixing and centrifugation (12,000 × g, 10 min, 4 °C). The supernatant was diluted to 53% methanol with water and centrifuged again to remove residual particulates. Chromatographic separation was performed on a Hypersil Gold column (2.1 × 100 mm, 1.8 μm) with a flow rate of 0.2 mL/min, using 0.1% formic acid in water (positive mode) or 5 mM ammonium acetate (negative mode) as mobile phase A and methanol as mobile phase B. The gradient program consisted of 2% B (0–1.5 min), 2–100% B (1.5–4.5 min), 100% B (4.5–14.5 min), and 100–2% B (14.5–15.5 min), followed by re-equilibration at 2% B (15.5–18 min). Mass spectrometry was performed using the following parameters: spray voltage, 3.5 kV; sheath gas, 35 psi; capillary temperature, 320 °C; S-lens RF level, 60; and auxiliary gas, 10 L/min at 350 °C. Metabolites were considered differentially abundant when the adjusted *p*-value was <0.05 (Benjamini–Hochberg method). Notably, pooled QC samples were regularly injected throughout the analytical sequence to monitor instrument stability, and standardized data pre-processing procedures were applied to ensure reproducibility and data quality. All blood and fecal samples from the study participants were collected on the same day.

### Mediation analysis of microbe–metabolite–clinical trait interactions

We performed mediation analysis to examine microbe–metabolite–clinical trait interactions using the R package mediation (version 4.5.1). This analysis employed a three-step causal framework to evaluate the following: (1) the association between microbial abundance and metabolite levels (path a), (2) the relationship between metabolites and blood pressure while adjusting for microbial abundance (path b), and (3) the total (path c) and direct effects (path c’) of microbes on blood pressure. All models were adjusted for age, sex, BMI, and batch effects, with false discovery rate (FDR) correction for multiple testing. Statistical significance of the mediation effects was assessed using 10,000 bootstrap iterations to calculate the average causal mediation effect (ACME) and 95% confidence intervals. We performed mediation analyses separately in the male and female cohorts, with adjustments for BMI and age.

### Statistical analyses

Alpha diversity metrics, including Chao1 richness, observed features, Simpson diversity index, and Shannon diversity index, were computed at the genus or species level using QIIME2. Beta diversity was assessed using principal coordinates analysis (PCoA) based on Bray–Curtis dissimilarity matrices, implemented in R using the vegan package (version 2.5–7). Differences in microbial community structure were statistically evaluated using analysis of similarities (Adonis) with 999 permutations. For biomarker discovery, the Wilcoxon rank-sum test and linear discriminant analysis effect size (LEfSe) were performed, with false discovery rate (FDR) correction using the Benjamini–Hochberg method. Associations between microbial features and clinical parameters were examined using Spearman correlation analysis in R.

## Results

### Characteristics of the participants

We recruited a total of 200 participants, including 100 male and 100 female individuals. Among these, 100 were hypertensive and 100 were non-hypertensive. The participants’ age, sex, BMI, SBP, DBP, and other indicators were recorded (Table 1). In our cohort, neither male nor female participants showed significant differences in terms of age (Female: *p* = 0.66; Male: *p* = 0.722) and BMI distribution (Female: *p* = 0.225; Male: *p* = 0.848). However, significant differences were observed in SBP (Female: *p* = 6.31E-15; Male: *p* = 1.63E-34) and DBP (Female: *p* = 5.63E-14; Male: *p* = 8.79E-17). Furthermore, in the female cohort, triglyceride (TG, *p* = 0.028), high-density lipoprotein (HDL, *p* = 0.1.69E-04), and fasting blood glucose (FBG, *p* = 0.006) levels showed significant differences between the patient and control groups. In the male cohort, significant differences were observed in TG (*p* = 0.024) and HDL (*p* = 0.007) levels.

**Table 1:** Characteristics of the participants.

Clinical characteristics	Female (*n* = 100)			Male (*n* = 100)		
	Case (*n* = 50)	Control (*n* = 50)	*P*-value	Case (*n* = 50)	Control (*n* = 50)	*P*-value
Age, y	57.6 ± 8.89	56.74 ± 10.51	0.66	54.76 ± 11.21	55.48 ± 8.83	0.722
BMI, kg/m^2^	24.77 ± 5.12	23.73 ± 3.16	0.225	26.71 ± 3.72	26.83 ± 2.73	0.848
SBP, mm Hg	150.49 ± 24.89	112.02 ± 6.38	6.31E-15	150.44 ± 10.52	113.78 ± 6.29	1.63E-34
DBP, mm hg	90.04 ± 13.74	69.88 ± 6.61	5.63E-14	89.82 ± 10.47	71.7 ± 6.05	8.79E-17
TG, mmol/L	1.97 ± 1.922	1.31 ± 0.81	0.028	1.91 ± 1.28	1.43 ± 0.71	0.024
TC, mmol/L	4.75 ± 0.96	4.90 ± 0.85	0.402	4.45 ± 0.67	4.62 ± 0.78	0.249
HDL, mmol/L	1.25 ± 0.36	1.54 ± 0.35	1.69E-04	1.10 ± 0.27	1.27 ± 0.32	0.007
LDL, mmol/L	3.06 ± 0.74	2.98 ± 0.71	0.588	2.85 ± 0.63	2.96 ± 0.68	0.385
FBG, mmol/L	5.83 ± 2.11	4.93 ± 0.74	0.006	5.38 ± 1.53	5.03 ± 0.90	0.169

The data for age, BMI, SBP, DBP, TG, TC, HDL, LDL, TG, and TC were presented as mean ± SD and compared using a *t*-test. BMI, Body Mass Index; SBP, Systolic Blood Pressure; DBP, Diastolic Blood Pressure; HDL, High-Density Lipoprotein; LDL, Low-Density Lipoprotein; TG, Triglycerides; TC, Total Cholesterol; FBG, Fasting Blood Glucose.

### Comparison of the gut microbiota between the hypertensive patients and controls at the genus level

**Figure 1:**
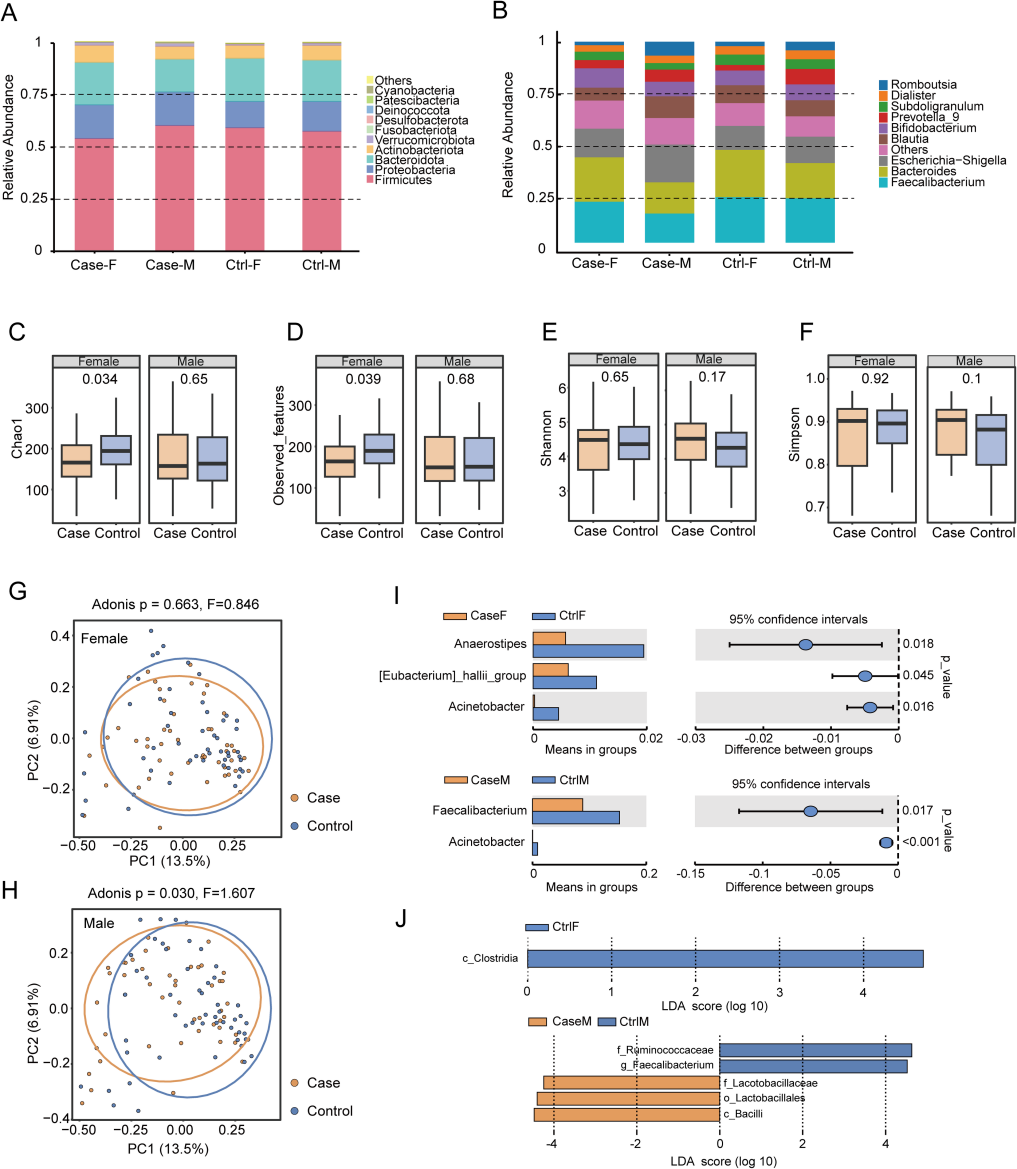
Comparison of the gut microbiota between the hypertensive patients and controls at the genus level. **(A)** Microbial composition at the phylum level. **(B)** Microbial composition at the genus level. **(C–F)** Comparison of alpha diversity between the case and control groups. **(G,H)** Comparison of beta diversity between the case and control groups. **(I)** Differential microbiota identified by the Wilcoxon rank-sum test. **(J)** Differential microbiota identified by LEfSe analysis.

To identify sex-associated gut microbial signatures in patients with hypertension, we used 16S rRNA sequencing to analyze data from a large cohort of 200 individuals. At the phylum level, the gut microbiota of both hypertensive and normotensive individuals were predominantly composed of Firmicutes, Proteobacteria, and Bacteroidota (Figure 1A). We did not observe statistically significant differences in the F/B ratio between hypertensive and normotensive individuals in either male or female cohort (Supplementary Figure S1A). At the genus level, *Bacteroides*, *Escherichia-Shigella*, and *Akkermansia* emerged as the dominant genera (Figure 1B).

Subsequent comparison of alpha diversity by sex revealed that the female hypertensive individuals exhibited significantly lower microbial richness than their normotensive counterparts, as indicated by Chao1 (*p* = 0.034) (Figure 1C) and observed features indices (*p* = 0.039) (Figure 1D). In contrast, no significant differences were observed in the male cohort for these metrics (Chao1: *p* = 0.65; observed features: *p* = 0.68) (Figures 1C,D). The analysis of Shannon (female: *p* = 0.65; male: *p* = 0.17) and Simpson indices (female: *p* = 0.92; male: *p* = 0.1) indicated no significant alterations in overall microbial diversity or evenness between hypertensive and normotensive individuals of either sex (Figures 1E,F).

Beta diversity assessment based on Bray–Curtis dissimilarity demonstrated that the female hypertensive patients showed no significant differences in gut microbial composition compared to the female normotensive individuals (*p* = 0.663, *F* = 0.846) (Figure 1G), whereas significant divergence was observed in the male participants (*p* = 0.030, *F* = 1.607) (Figure 1H).

### Identification of potential sex-associated microbial biomarkers at the genus level

To further identify differentially abundant taxa, the Wilcoxon rank-sum test revealed significant depletion of *Anaerostipes* (*p* = 0.018), [*Eubacterium*]*_hallii*_group (*p* = 0.045), and *Acinetobacter* (*p* = 0.016) in the female hypertensive participants (Figure 1I). In the male participants, *Faecalibacterium* (*p* = 0.017) and *Acinetobacter* (*p* < 0.001) were significantly reduced. LEfSe analysis corroborated these findings, identifying a significant decrease in *Clostridia* among the female participants and reconfirming *Faecalibacterium* depletion in the male participants (Figure 1J). Collectively, these results highlight *Faecalibacterium* as a consistent microbial signature, potentially serving as a male-associated gut microbiota biomarker in hypertension.

### Comparison of the gut microbiota between the hypertensive patients and controls at the species level

**Figure 2:**
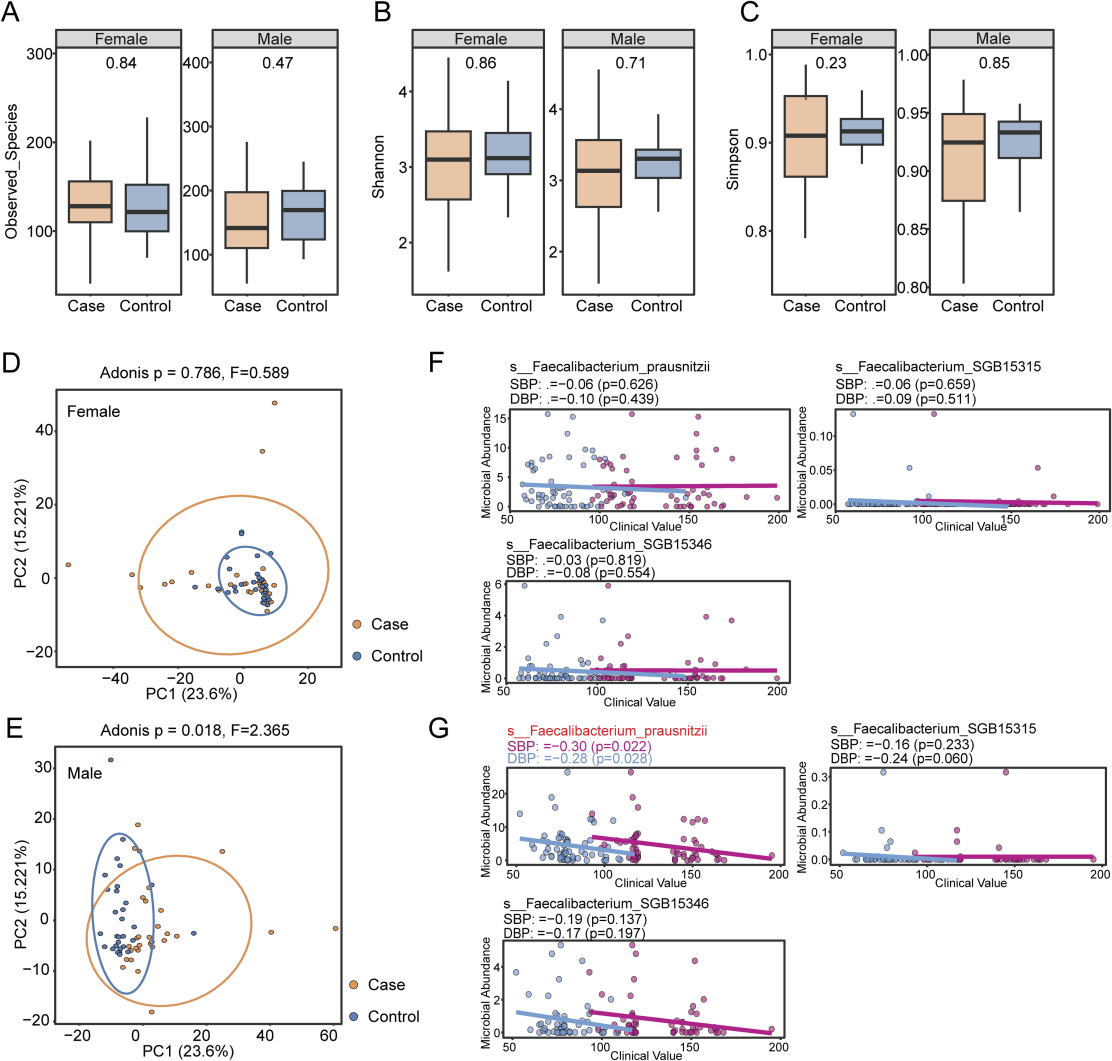
Comparison of the gut microbiota between the hypertensive patients and controls at the species level. **(A–C)** Comparison of alpha diversity between the case and control groups. **(D,E)** Comparison of beta diversity between the case and control groups. **(F)** Associations between SBP, DBP, and *Faecalibacterium* species in the female population. **(G)** Associations between SBP, DBP, and *Faecalibacterium* species in the male population.

To further identify microbial biomarkers at the species level, we randomly selected 120 samples (60 male and 60 female, with 30 hypertensive and 30 normotensive individuals in each sex group, clinical characteristics are detailed in Supplementary Table S2) for metagenomic sequencing. Notably, we did not observe statistically significant differences in the F/B ratio between the hypertensive and normotensive individuals in either sex group (Supplementary Figure S1B). Alpha diversity analysis at the species level revealed no significant differences between the hypertensive and normotensive individuals in either sex group for Observed species (female: *p* = 0.84; male: *p* = 0.47) (Figure 2A), Shannon index (female: *p* = 0.86; male: *p* = 0.71) (Figure 2B), or Simpson index (female: *p* = 0.23; male: *p* = 0.85) (Figure 2C). Beta diversity assessment using Bray–Curtis dissimilarity showed no significant compositional differences in the female individuals (*p* = 0.786, *F* = 0.589) (Figure 2D), whereas the male hypertensive patients exhibited distinct microbial profiles compared to the controls (*p* = 0.018, *F* = 2.365) (Figure 2E).

### Identification of sex-associated microbial biomarkers at the species level

Building upon our previous identification of *Faecalibacterium* as a potential biomarker, metagenomic analysis identified three *Faecalibacterium* species (Supplementary Table S3): *F. prausnitzii*, *F. SGB15315*, and *F. SGB15346*. Notably, only in the male participant did *F. prausnitzii* show significant negative correlations with both systolic (Figure 2G) (*R* = − 0.30, *p* = 0.022) and diastolic blood pressure (*r* = −0.28, *p* = 0.028). No significant associations were observed for the other two species in the male cohort (*F. SGB15315*: SBP *r* = −0.16, *p* = 0.233; DBP *r* = −0.24, *p* = 0.060; *F. SGB15346*: SBP *r* = −0.19, *p* = 0.137; DBP *r* = −0.17, *p* = 0.197). In female participants, none of the *Faecalibacterium* species demonstrated significant correlations with blood pressure parameters (Figure 2F) (*F. prausnitzii*: SBP r = −0.06, *p* = 0.626, DBP *r* = −0.10, *p* = 0.439; *F. SGB15315*: SBP *r* = 0.06, *p* = 0.659, DBP *r* = 0.09, *p* = 0.511; *F. SGB15346*: SBP *r* = 0.03, *p* = 0.819, DBP *r* = −0.08, *p* = 0.554). These results further substantiate *F. prausnitzii* as a male-associated microbial biomarker for hypertension. In addition, significant negative correlations with triglyceride (TG) levels were observed only in the male cohort, specifically for *F. prausnitzii* and F. SGB15346 (Supplementary Figure S2).

### Functional characterization of the gut microbiome

**Figure 3:**
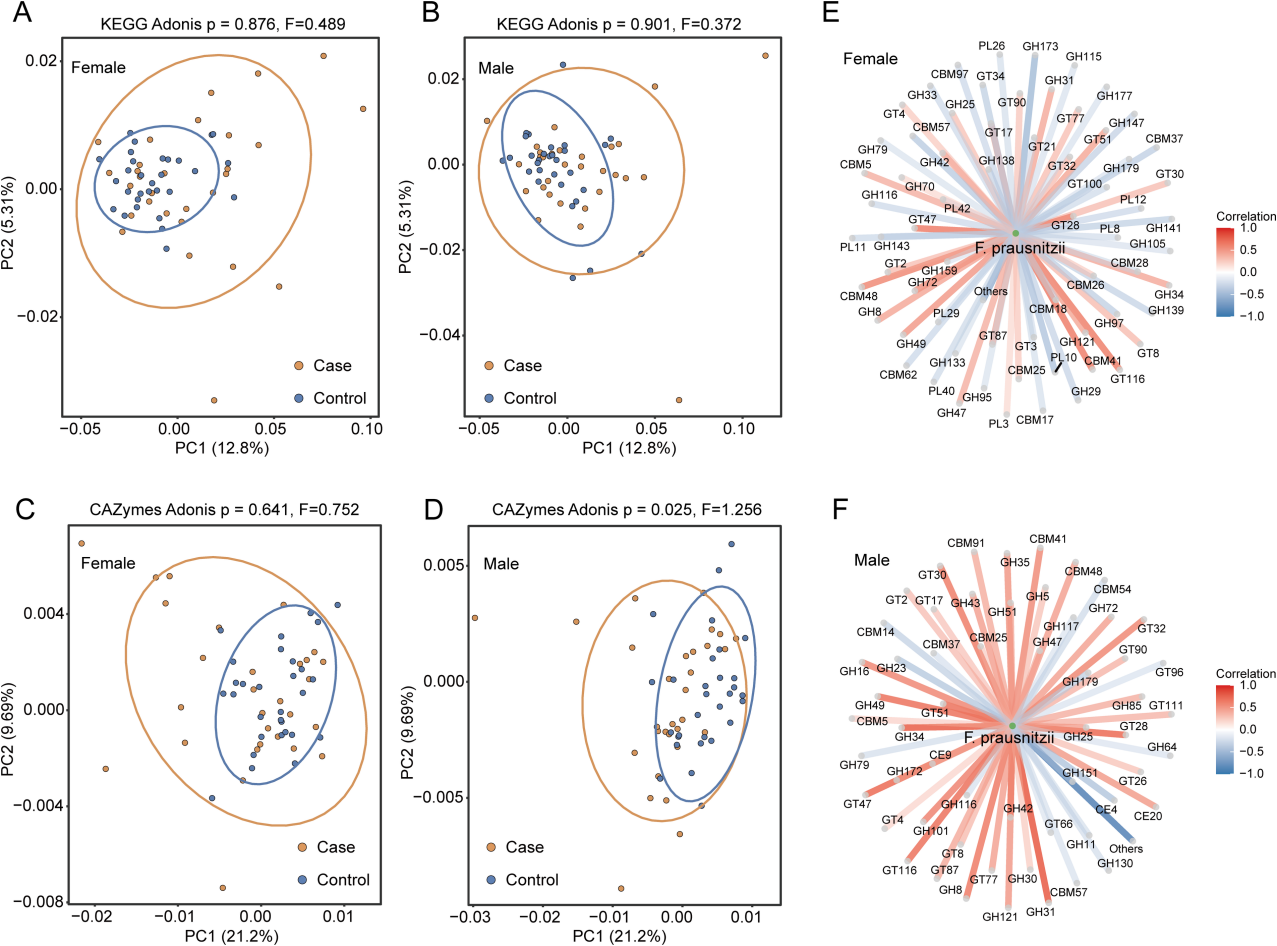
Functional characterization of the gut microbiome. **(A,B)** Comparison of beta diversity between the case and control groups based on KEGG composition. **(C,D)** Comparison of beta diversity between the case and control groups based on CAZymes composition. **(E)** Associations between CAZymes and *Faecalibacterium* species in the female population. **(F)** Associations between CAZymes and *Faecalibacterium* species in the male population.

To further elucidate the functional implications of the gut microbiota in hypertension, we performed KEGG pathway annotation. However, no significant differences in overall functional profiles were observed between the hypertensive and normotensive individuals in either sex group (female: *p* = 0.876, *F* = 0.489; male: *p* = 0.901, *F* = 0.372) (Figures 3A,B). Given previous reports suggesting that *F. prausnitzii* may regulate blood pressure through short-chain fatty acid (SCFA) metabolism, we specifically analyzed carbohydrate-active enzyme (CAZyme) gene composition. While no significant differences were detected in the female individuals (*p* = 0.641, *F* = 0.752) (Figure 3C), the male hypertensive patients exhibited distinct CAZyme profiles compared to the controls (*p* = 0.025, *F* = 1.256) (Figure 3D). Subsequent correlation analysis revealed stronger positive associations between *F. prausnitzii* abundance and carbohydrate metabolism genes in the male participants (Figures 3E,F), supporting the hypothesis that *F. prausnitzii* may influence blood pressure regulation through the modulation of carbohydrate utilization pathways, consistent with previous mechanistic reports.

### Mediation analysis of microbe–metabolite–clinical trait interactions

**Figure 4:**
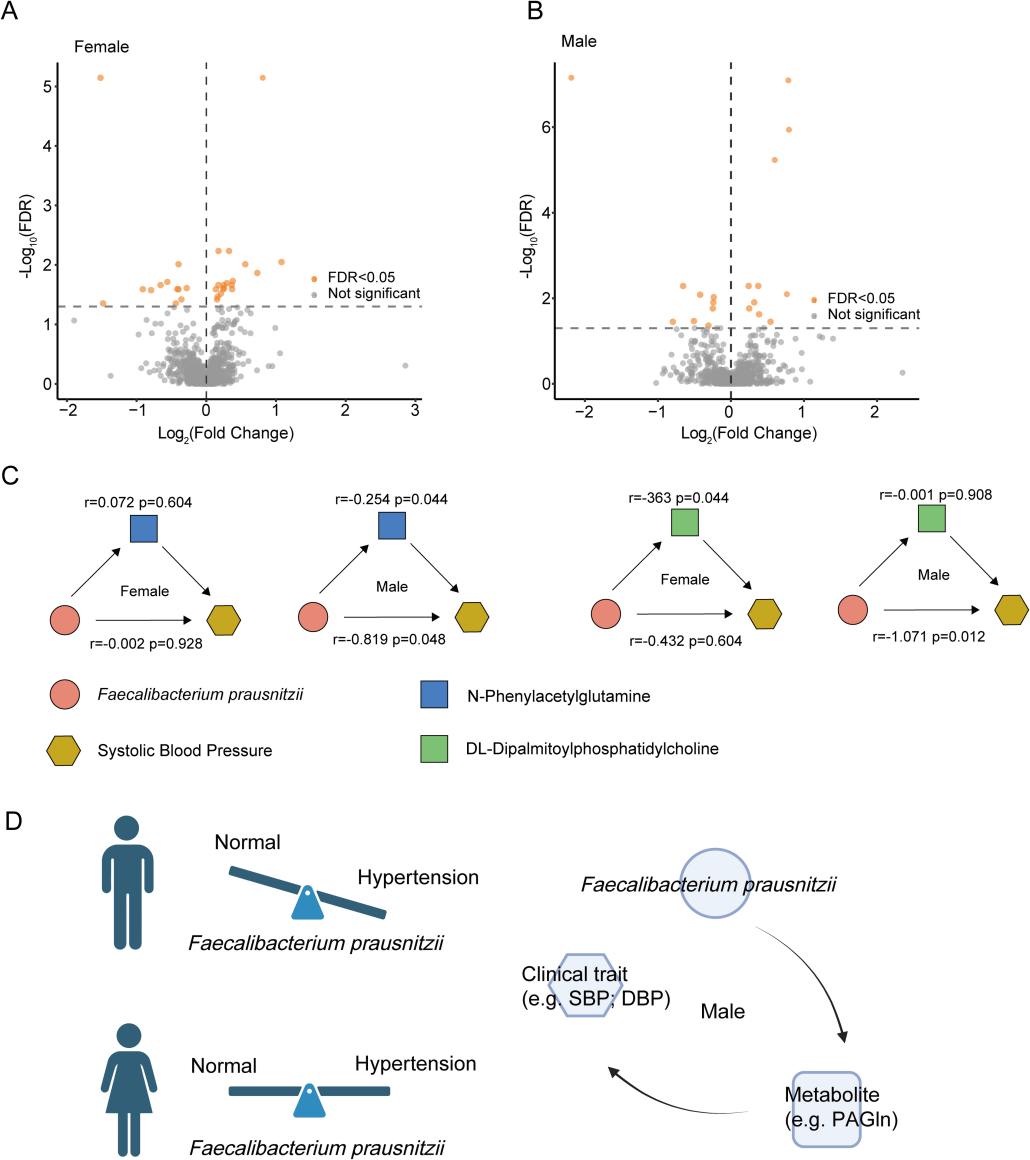
Functional characterization of the gut microbiome. **(A,B)** Differential serum metabolites between the case and control groups. **(C)** Mediation analyses to determine associations between *F. prausnitzii* and blood pressure. **(D)** Sex-associated mechanism diagram.

Beyond the well-documented role of short-chain fatty acids, we sought to identify additional serum metabolites potentially involved in blood pressure regulation by performing untargeted metabolomic profiling using UHPLC–MS on all participants. Sex-stratified analysis revealed distinct metabolic signatures: In the female participants, we identified 30 differentially abundant metabolites (18 upregulated and 12 downregulated in the hypertensive individuals) (Supplementary Table S4; Figure 4A), while the male participants exhibited 23 altered metabolites (11 upregulated and 12 downregulated; Supplementary Table S4; Figure 4B). To establish gut microbiota–serum metabolite–clinical phenotype relationships, we conducted mediation analyses to determine whether *F. prausnitzii* influences blood pressure through metabolic regulation. Intriguingly, in the male participants, *F. prausnitzii* demonstrated both direct (*r* = −0.819, *p* = 0.048) and N-Phenylacetylglutamine-mediated indirect effects on systolic blood pressure (SBP; *r* = −0.254, *p* = 0.044) (Supplementary Table S5; Figure 4C), whereas neither pathway reached significance in the female participants (direct: *r* = 0.002, *p* = 0.928; indirect: *r* = 0.072, *p* = 0.604). Notably, we uncovered a novel female-associated mechanism in which *F. prausnitzii* was associated with reduced SBP through downregulation of DL-Dipalmitoylphosphatidylcholine (*r* = −0.363, *p* = 0.044) without direct effects (*r* = −0.432, *p* = 0.604) (Supplementary Table S5; Figure 4C), while the male participants showed only direct associations (*r* = −1.071, *p* = 0.012) without mediation (*r* = −0.001, *p* = 0.908). These findings collectively suggest novel mechanistic pathways through which *F. prausnitzii* may exert antihypertensive effects via the modulation of specific serum metabolites in a sex-dependent manner (Figure 4D).

## Discussion

Hypertension represents a major modifiable risk factor for the global cardiovascular disease burden, yet approximately 30–50% of patients exhibit poor blood pressure control despite current therapies (NCD Risk Factor Collaboration, 2021). The gut microbiome has emerged as a novel therapeutic target to treat hypertension (Ganesh et al., 2018; O’Donnell et al., 2023). Our study advances this field by providing a multi-omics characterization of sex-associated gut microbiota signatures in a well-phenotyped Xinjiang cohort, integrating 16S rRNA sequencing, shotgun metagenomics, and serum metabolomics to establish mechanistic links between microbial taxa and blood pressure regulation.

The relationship between gut microbiome alpha diversity and hypertension has been characterized by inconsistent findings in previous studies (Cai et al., 2023). This heterogeneity may largely stem from the common oversight of sex-associated differences and the masking effect of geographically diverse cohorts. Our investigation, conducted within the unique biogeographical context of Xinjiang, controlled for these factors and thereby uncovered subtle yet distinct sex-driven disparities in alpha diversity. While large-scale studies (Louca et al., 2021) have reported significant gut microbiome alterations in female individuals with hypertension, our findings from a specific Xinjiang cohort refine this perspective. We reveal that within this unique population, the most discernible microbial shifts are not observed in female individuals but are instead prominent in male individuals. Future studies are needed to determine whether this sex-associated pattern exists in other populations.

A key finding from our results is the identification of *F. prausnitzii* as a male-associated biomarker, showing significant negative correlations with systolic blood pressure. Previous studies have reported that SCFAs can regulate blood pressure (Gomez-Arango et al., 2016; Huart et al., 2019; Lan et al., 2022). Among all butyrate producers, *F. prausnitzii* is the most abundant in fecal samples, with its proportion reaching 13–17.6% (Singh et al., 2022). We also observed a reduced abundance of *Clostridium* in the gut of the female individuals with hypertension. We hypothesize that this decline, potentially driven by a decrease in SCFA-producing species such as *Clostridium butyricum* (Luo et al., 2023; Zhao et al., 2020), may contribute to elevated blood pressure. Furthermore, we propose that sex hormones (e.g., estrogen) modulate the gut microenvironment, leading to these sex-associated shifts in *Clostridium* abundance. The reduction observed in the female hypertensive individuals may thus represent a disruption of a protective, SCFA-mediated metabolic pathway. In our study, although we did not specifically focus on SCFAs, given their well-documented associations in previous literature, we uncovered a correlation between *F. prausnitzii* and CAZymes. This finding provides compelling evidence supporting the aforementioned mechanisms of microbe-mediated blood pressure regulation.

Our findings highlight the importance of investigating alternative microbial metabolites in hypertension pathophysiology. Specifically, we identified N-Phenylacetylglutamine (PAGln) as a novel mediator of *F. prausnitzii*’s antihypertensive effects in male individuals, suggesting distinct sex-associated mechanisms beyond classical SCFA pathways. The *F. prausnitzii*–PAGln–blood pressure axis likely functions through multiple interconnected biological pathways, which are increasingly recognized in cardiovascular research. Emerging clinical evidence demonstrates that plasma PAGln levels serve as an independent predictor of adverse cardiovascular outcomes, including myocardial infarction and stroke (Wei et al., 2023; Zong et al., 2022). This association appears to be mediated through PAGln’s interaction with G protein-coupled receptors, particularly adrenergic receptors (Nemet et al., 2020), which may explain its hypertensive effects. Mechanistic studies suggest that PAGln promotes platelet hyperreactivity and enhances thrombosis potential (Nemet et al., 2020; Fang et al., 2022; Huynh, 2020). These pro-thrombotic effects could contribute to elevated blood pressure through microvascular occlusion and subsequent endothelial dysfunction.

*F. prausnitzii* is considered a promising next-generation probiotic (Al-Fakhrany and Elekhnawy, 2024; Martin et al., 2017). The sex-associated reduction of *F. prausnitzii* observed in the male hypertensive individuals in our study highlights it as a compelling target for precise microbial intervention. Future clinical trials are essential to investigate the safety, colonization efficacy, and anti-hypertensive potential of *F. prausnitzii* supplementation, paving the way for microbiome-based precision medicine in cardiovascular disease.

Several limitations should be considered when interpreting our findings. Although our mediation analyses suggest plausible mechanistic pathways, longitudinal or interventional studies are needed to confirm these associations. Second, the study population was drawn from a single geographic region (Xinjiang), which may limit the generalizability of the findings to other ethnic groups with distinct dietary patterns or genetic backgrounds. Third, although we adjusted for major confounding factors including age, BMI, and medication use, residual confounding from unmeasured variables (e.g., detailed dietary intake, physical activity levels) cannot be excluded. Fourth, the sample size, while adequate for initial discovery, may have limited power to detect more subtle sex-associated effects, particularly for subgroup analyses.

Collectively, our findings initially delineate the microbe–metabolite–clinical trait axis underlying sexual dimorphism in hypertension pathogenesis, providing unprecedented mechanistic insights into sex-associated host–microbiome crosstalk.

## Data Availability

The datasets presented in this study can be found in online repositories. The names of the repository/repositories and accession number(s) can be found in the article/Supplementary material.
