# Supplementary material for: Sex-specific association between gut Faecalibacterium prausnitzii and hypertension in male individuals
Source: Front Microbiol. 2025 Dec 17;16:1683587. doi: 10.3389/fmicb.2025.1683587 (PMC12753911; doi:10.3389/fmicb.2025.1683587)
Supplement: Supplementary file 1 [file Data_Sheet_1.docx]

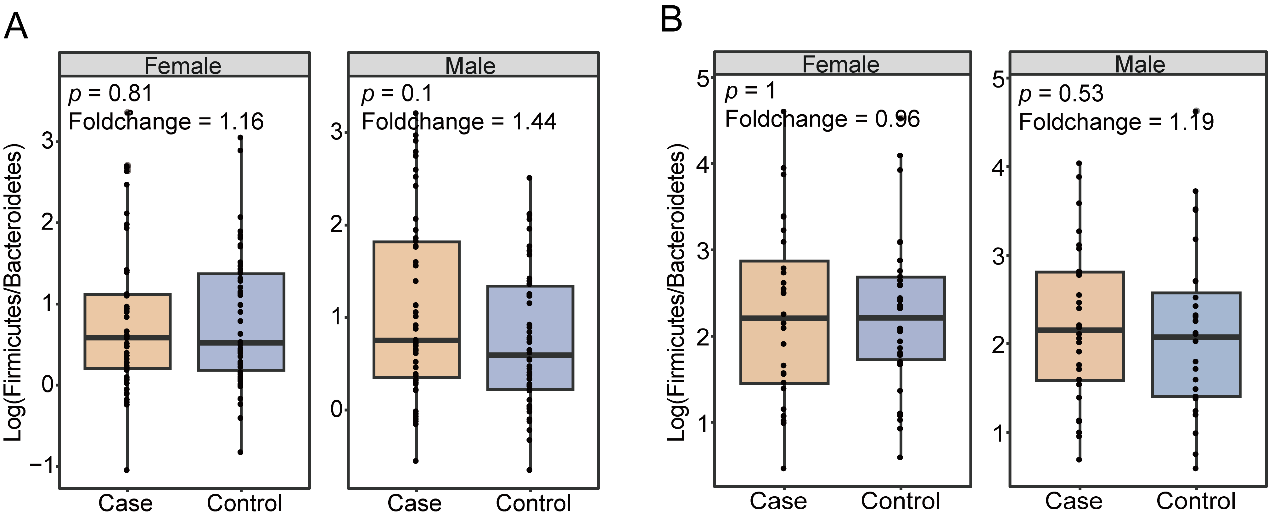


**Figure S1. Comparison of the F/B ratio between males and females in both 16S rRNA sequencing (A) and metagenomic sequencing data (B).**


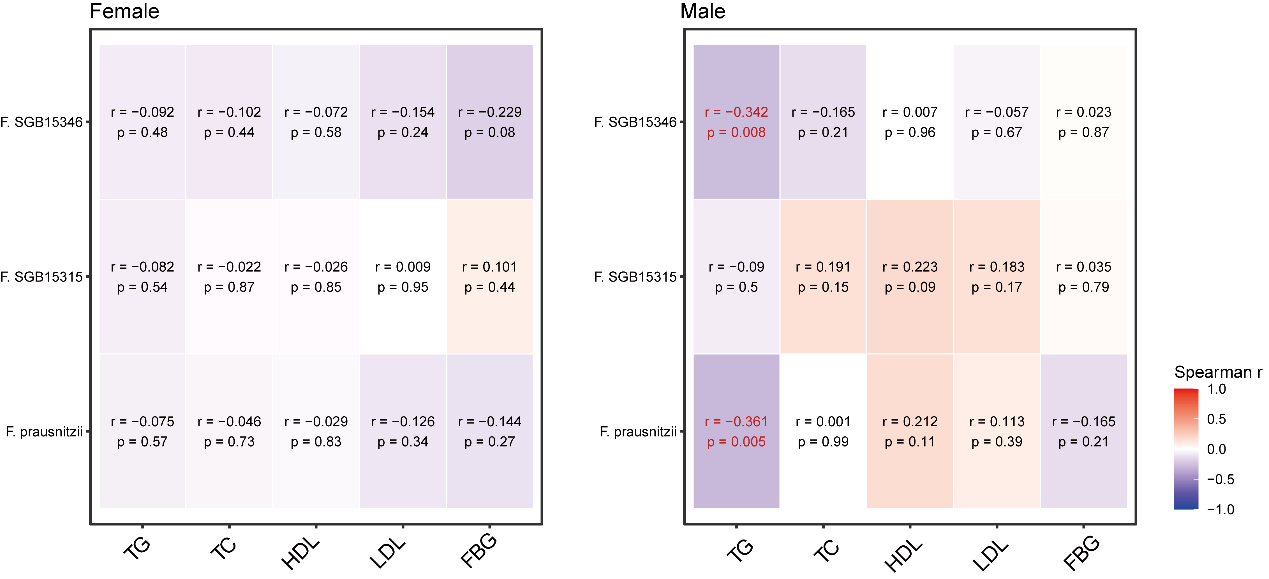


**Figure S2. Spearman correlations between three *Faecalibacterium* species and clinical indicators in hypertensive patients, stratified by sex.**
